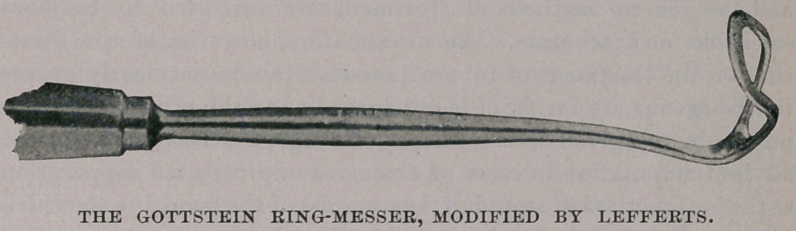# Adenoma of the Naso-Pharynx in Relation to Bronchitis and Asthma1Read before the Section on Medicine of the Buffalo Academy of Medicine, April 11, 1893.

**Published:** 1893-05

**Authors:** Horace Clark

**Affiliations:** (Harv. A. B., M. D.) of Buffalo; Surgeon Charity Eye, Ear, and Throat Hospital; formerly Clinical Assistant Throat Department College Physicians and Surgeons, New York; Fellow of the Massachusetts Medical Society, etc.; 21 West North Street


### Buffalo Medical and Surgical Journal

Vol. XXXII. MAY, 1893. No. 10.

### Original Communications.

### ADENOMA OF THE NASO-PHARYNX IN RELATION TO BRONCHITIS AND ASTHMA.[1]

By HORACE CLARK, (Harv. A. B., M. D.,) of Buffalo.

Surgeon Charity Eye, Ear, and Throat Hospital ; formerly Clinical Assistant Throat Department College Physicians and Surgeons, New York ; Fellow of the Massachusetts Medical Society, etc.

In the consideration of this subject I claim no originality, further than the report of a few of my own cases by way of typical illustration ; for a few suggestions ; and for certain statements which I wish especially to emphasize.

The compiler's work is, however, justifiable ; for, consulting the best and most recent authorities, he gives to the general practitioner, or to the specialist in another department, a connected whole upon a given subject. Especially is this true when the subject is one contained in monograph literature, rather than in a recognized special text-book. Furthermore, an original investigation by others is very often invited from his work. It is to him, frequently, that our thanks are due for transforming into strong and indisputable argument, that which has been previously so fragmentary as to be considered merely theoretical or illusory. If I did not hope to fulfil this definition : to give you some positive, absolutely new information upon my subject, I should not have accepted this date for a paper.

"Adenoids" is a term understood by the merest tyro in medicine to mean a certain pathological condition in the naso-pharynx, responsible, in some cases—happily few, now that the condition is recognized,—for years of suffering on the part of the patient, and years of anxiety on the part of the parents. So far as this term goes for the *name of a condition*, I do not know but it serves as well as any other. I cannot understand, however, how the already

1. Read before the Section on Medicine of the Buffalo Academy of Medicine, April 11, 1893.

enormous amount of literature upon the physiology and pathology of this condition can be regarded as but useless, if it has been written with the idea that this superabundant growth is really adenoid tissue. *It is nothing of the kind*, gentlemen, unless I am very much misinformed as to the nature of *adenoid* tissue.

I find in a volume by Jurasz[1], University of Heidelberg, almost fresh from the press, the statement that adenoid tissue, adenoma of the naso-pharynx, and hypertrophy of the pharyngeal tonsil, are synonymous terms. On the other hand, I find in Ziegler's Pathology[2] the statement that an adenoma is a tumor constructed after the type of a secreting gland ; that for a given tumor to be entitled to that name it must consist of epithelial elements on the one hand, and of vascular connective tissue on the other. "It is a true neoplasm, characterized, *physiologically*, by its impotence to produce the normal gland secretion" (upon which type it is constructed), " *anatomically* by its want of relation to the tissue in which it is seated. Its consistence, color, and structure all mark it off plainly from the surrounding tissue. A gland enlarged by overgrowth, or overwork, or chronic inflammation, cannot be described as an adenoma. It is a hyperplasia, a production of lymph-nodules ; and if it be the true gland tissue which is excessively developed, and not merely the fibrous framework, the physiological activity of the gland is thereupon increased." Without quoting, I will mention Orth[3] and Eichorst[4] to the same effect. In any of the works upon microscopical anatomy[5] which I possess, I cannot find this tissue, which is removed from the naso-pharynx, designated as adenoid. Either we must admit there is no such thing as this conglomeration of glands in the vault of the pharynx, constituting, if you please, the so-called pharyngeal tonsil ; that these glands do not undergo a process of hyperplasia, and that lymph tissue *does* occur between the fibrous interstices, or, we must seek for some new explanation of the structure of the growth which we find in this situation. For, *adenoid tissue, or tumor*, it *certainly* is not.

In order to explain etiological factors as relating, especially, to age ; to explain the origin of certain conditions in the lower portions of the air-passages, and in the lungs ; in short, in order for there to be a reason for this paper, I must define the condition somewhat as follows :

1. A. Jurasz : Krankheiten der Oberen Luftwege, 1892.
2. Ernst Ziegler: Path. Anat., Eng. Ed., 1885.
3. Orth: Lehrbuch der Spec. Path. Anat., 1887.
4. Eichorst: Spec. Path. und Therapie.
5. Schiefferdecker u. A. Kossel: Gewebelehre d. Mensch. Körp. Two vols., 1891.

It is an outgrowth of the mucous membrane, in which the glands are simultaneously enlarged. Lymphoid tissue is produced in the form of diffuse cell-infiltration of the mucous membranes—in other words, as lymph-follicles, or lymph-nodules.

Having made myself clear on this point, I shall use the term adenoids throughout this paper as a matter of convenience.

First, as to the relation of this glandular hyperplasia to bronchitis.

The upper part of the thorax, upon which the large auxiliary muscles of inspiration are inserted, becomes inordinately spread apart in consequence of the forced breathing incident to the obstructive lesion in the vault of the pharynx ; while the lower parts, in consequence of the increased intra-thoracic negative pressure, becomes compressed, partly by the diaphragm being drawn upwards, partly, too, by the narrowing of the lower intercostal spaces. This form of the thorax occasions an emphysematous enlargement of the alveoli of the lungs, especially in their upper anterior portions. Lower down the condition is one closely resembling atelectasis. Acute or chronic bronchitis is but a step removed.

I wish to mention, in passing, that Loewenberg[1] has made some very interesting observations and studies upon this peculiar anatomical change in the thorax, as entirely distinct from that form known as " pigeon-breast," of rachitic origin. His investigations are a model of ingenious scientific research.

Another way in which bronchitis may occur is borne out by the clinical facts which I meet with in my daily practice, that these enlarged lymph-follicles upon the pharynx, especially when they extend up behind the soft palate, are a prolific source of cough. Examine the upper parts of the lungs, and you will invariably find, if the condition has persisted for any length of time, a greater or less degree of bronchitis. If the germ theory of bronchitis be correct, then in the bronchitis accompanying *this* condition the germ must reside in these follicles, and should be easy of microscopical demonstration. For, again and again, by means of the cautery point, I have completely stopped a cough of months—in several cases which I call to mind, of years—duration, the history being one not of constant cough, but of cough and oppression in the chest, coming on immediately after a sore throat, to which, says the patient, "I seem to be predisposed, without, indeed, having at the same time cold in the head." This condition is of

1. Loewenberg : Des Végétations Adénoïdes, etc , *Ex. du Jour. de Thérap.*, 1881.

too common occurrence in every throat specialist's practice to warrant the citation of cases.

What has this to do with adenoids? Simply this, that where these lymph-nodules are seen upon use of the tongue depressor, they are invariably found in children by means of the rhinoscope more or less markedly developed in the vault of the pharynx. Fraenkel[1] gives it as his opinion that the follicular pharyngitis of the adult is nothing but the persistence and continuation of the same condition in the child.

That adenoids are causative of bronchitis, even when not of sufficient size to be an obstructive lesion in the upper air-passages, is due to a morbid condition in the naso-pharynx, present also, perhaps, in the nose, giving rise to (1) circulatory disturbance; (2) disturbances of the motor-nerve tracts. As to the way in which the former may operate, I have to report that both the arterial supply and the venous outlet of the upper air-passages and the bronchial tubes are regulated by the same motor tracts. (This explanation is entirely new, and is due to the untiring research of Dr. F. H. Bosworth.[2]) The influence of the latter must be immediately admitted, I think, by the mere mention of the pneumogastric nerve, its branches, and branches of communication, to say nothing of the trigeminus as representing also, in part, the nerve supply.

(The part played by the trigeminus nerve is a subject about which I have had some little experience from puncture experiments. I hope, at no distant date, to give you some definite information on this point.)

It is supposed that the hyper-secretion attendant upon adenoids is passed on into the larynx, giving rise to co-existent inflammation there, which extends downwards, and it is, therefore, offered as a cause of bronchitis. I cannot agree with this. For, why does not the normal secretion from the retro-nasal space, poured out in a daily quantity of several drachms (if I remember rightly), cause laryngitis? This normal secretion is *swallowed*. So, too, I believe the abnormal secretion is either swallowed or hawked out, and does not pass on into the larynx. For illustration, why does not the hemorrhage (sometimes considerable), incident to operation, get into the larynx? If it does not flow out of the

1 B. Fraenkel: *Sep. Ab. Deutsch. Med. Wochen.*, No. 41 ff., 1884. Ueber Aden. Veg.

2. Treatise on Diseases of the Nose and Throat, 1892. Vol. I.

mouth, it appears with the first attack of vomiting in recovery from the narcosis.

I would call attention to the fact that there is a distinction, clinically, between catarrhal inflammation of the naso-pharynx and that of the intra-nasal passages. Nasal lesions which are productive, mechanically, both of bronchitis and asthma, are very different from adenoids, in this sense, in their general course and symptomatology.

I give vaso-motor circulatory disturbance as one factor in explanation of bronchitis occurring in the presence of adenoids. Let me anticipate a question on this point by reminding you that this hypertrophied glandular tissue covers, oftentimes, the whole extent of the basilar process of the occipital bone, spreading over the whole width of the vault to the fossa of Rosenmüller, and even as far as the "cushion" at the entrance to the Eustachian tube. Furthermore, the local mucous membrane of the part does not essentially differ from that of the whole respiratory tract. We find tubular and racemose glands, the whole upper surface of the membrane being covered by ciliated epithelium.

In a monograph by the celebrated clinician, Kussmaul,[1] I find the statement that, an active hyperemia of the lungs, with its sequelæ, may very well be caused by an obstructive lesion in the upper air-passages.

It is not *necessary* that bronchitis should follow measles. That it is one of the sequelæ in many cases, may be dependent upon the existence, known or unknown, of adenoids. On this point I have had no clinical experience; nor can I find any cases recorded. I mention this simply for what it is worth, and for information. That the acute exanthemata aggravate the condition; that, indeed, they often start into rapid growth these glands which before have been quiescent, is a well-recognized clinical fact.

Everyone recognizes the importance of pure air in the prophylaxis of bronchitis. I refer, for example, to workers in factories where particles of dust are inhaled.[2] Given, also, an obstructive lesion in the upper air-passages, and fibroid-phthisis is only too often a consequence hastening the dire result.

The function of the nose as a respiratory organ is, undoubtedly, the most important, delicate, and hence intricate part of the whole air tract. Some sixteen ounces of water are poured out from the

---

1. Kussmaul : Zeit. für Rat. Med., 1865.
2. Cassells : "Shut Your Mouth and Save Your Life." Edin., 1877.

nose in the course of the day. Interfere with the supply of moisture to the inspired air; interfere with the air being brought to the temperature of the body, through the mechanism of the turbinated bodies, before passing on to the lungs, and you cause disturbances in the lungs, which are going to result in bronchial disease—a trivial factor in the general picture of the subject of an obstructive lesion in the upper air-passages.

Niemeyer[1] gives it as his clinical experience, that adenoids are often causative of attacks of false croup in children. Frequent repetition of these attacks results in bronchitis.

Other relations between adenoids and bronchitis might be mentioned. (For example, Tornwaldt's[2] cases of post-nasal obstruction, in which enlargement of the so-called "pharyngeal bur sa' figures conspicuously.) I think, however, that sufficient has been adduced by the references already named.

Among my own cases, I shall briefly mention two, illustrative not only of the proposition to be proved, but of the general picture presented by the subject of adenoid disease—a picture not often so deplorable as this one, and yet, in general outline, quite common. The case illustrates, also, the occasional necessity of differential diagnosis—a subject which the limit of this paper precludes.

Case I. C. O., came to me in New York, in January, 1891, with a diagnosis of incurable migraine, and incipient phthisis. The patient was a girl, sixteen years old, with a clear family history, and a personal history of no sickness except measles. She had never menstruated. For some two years her parents had noticed that she had been running down. During the six months previous to my examination, she had occasionally spit out bloody matter, without ever having had hemorrhage. There had been gradually increasing cough, with expectoration, more profuse in the morning. Night-sweats had recently come on, requiring two or three changes of clothing. Her hands were wet and cold. (Chattelier[3] says that these sweats are one of the most frequent and distressing symptoms of obstructive lesions of the retronasal cavity.) (Salter,[4] F. C. Shattuck,[5] Franklin Hooper,[6] and others, state the same.) Her sleep was greatly disturbed by a peculiar kind of snoring. Naturally a bright girl, she had been obliged to give up all study. The parents had noticed that after she had been asleep a few hours, notably when the snoring came on, she breathed with her

1. Paul Niemeyer: Atmiatrie, p. 86.
2. Tornwaldt: Ueber d. Bedeut. d. Bursa Pharyngea.
3. Henri Chatellier: Des Tumeurs Adénoïdes du Phar. Nasal., 1886.
4. Hyde Salter: Asthma, Path. and Treat., Lond., 1860.
5. Shattuck: Keating's Enc Children's Dis.
6. Hooper: Adenoid Veg. in Children, *Bost. Med. and Surg. Jour.*, March 15, 1888.

mouth open. During the day mouth-breathing had been noticed with increasing frequency. She said she could hear sounds in her lungs. Examination of the lungs gave me nothing but a chronic bronchitis of no considerable extent. There was a blowing systolic murmur, pretty generally distributed over the chest, almost disappearing after walking rapidly several times across the room. Not to detain you longer, the removal of adenoid tissue, so completely filling the vault of the pharynx as to be seen below the uvula, a portion of the overgrowth being pushed into the posterior nares, was followed within two months by a complete change in the girl's general condition.

Case II. A. B., a young woman of twenty years, was referred to me in February, 1893, by Dr. M. B. Folwell, of this city. The story was, that up to two years ago she had enjoyed such perfect health as to ride horse-back, play tennis, row boats, and walk miles. Since that time she has been gradually falling off. About six months since, she began to cough, especially in the morning. That "tired feeling" seemed alarmingly to increase. Kind (?) friends stated to her that, undoubtedly she had consumption. Brooding over this, occasioned an exacerbation in the decline. As her physician was also a warm friend since babyhood, she rather doubted his statements that she did not have consumption, thinking he meant to be kind. That the gentleman did think, although he did not so state to me, that there might be localized tuberculosis in the larynx, was evident from his manifest surprise and pleasure when I assured him there was no intra-laryngeal complication. The case was one of *bronchitis* with anemia, as, among other clinical findings, the hemoglobin percentage and blood-count showed. In etiology, it was a case of retro-nasal obstruction, of *forced breathing*, causing an improper exchange of gases in the lungs. The result of operation I give in the words of the physician: "The girl is literally made over."

Passing on to asthma occurring with adenoid disease.

Whether he is right, the clearest exposition which I have ever found of this most indefinite and entangled subject, is due to Dr. Bosworth,[1] to be found in two new volumes from his pen. Since he stated to me, a few days ago, that he had been horribly misquoted with regard to his views in this matter, I shall endeavor to quote him correctly. Bronchial asthma he considers apart from asthma with *neurosis*, and refers to text-books upon general medicine for exposition of the former.

Asthma is due to:

1. General neurotic condition.
2. Some obscure condition of the atmosphere.
3. Diseased condition of the upper air-passages.

1. Loc. cit.

Hay-fever is thus tabulated as to its etiology:

1. Neurotic habit.
2. Atmospheric conditions.
3. Disordered condition of the upper air-passages.

I interpret this to mean that both conditions are, therefore, due essentially to one and the same causes; except that asthma is a *development* which may or may not occur in the course of "hay-fever." Reflection upon this idea has occasioned my own definition, which I shall presently respectfully submit.

"Hay-fever" is designated as "*vaso-motor* rhinitis;" whereas, he calls the other condition "*perennial*" asthma.

Perennial asthma is defined as a "*disease* characterized by diurnal and seasonal recurrence of attacks of dyspnea, due to an obstruction in the bronchial tubes, in such a manner as to interfere with the free ingress and exit of air therefrom."

Bergson,[1] as far back as 1852, also characterized asthma as a *disease*. That it is a symptom and not a disease, is insisted upon among the earlier writers, notably by Burckhardt,[2] and within recent years by F. C. Shattuck.[3] Shattuck's point is, that if it is a disease, why does it so often disappear in children, for example, after puberty? Other writers state that, asthma has no pathological anatomy. In that case, I cannot see how it can be called a *disease*, except in the sense that dropsy is a disease. But Shattuck's objection to its being thus designated because it disappears, is hardly tenable. I would inquire if adenoid tissue in the vault of the pharynx is not to be considered a *disease* simply because it tends to disappear; when, in virtue of general bodily metamorphosis, a lymphatic temperament is changed to another, and this tissue begins to atrophy, finally as such to disappear?

The theory that asthma is due to bronchial spasm seems to have been abandoned by most of the younger writers of the present day.

I would suggest that if these paroxysmal attacks of dyspnea are due to bronchial spasm, why do they have such a predilection for the night as a time for occurrence? Is not undisturbed sleep directly opposed to any sort of spasm? In the discussion of this paper I shall surely be told by some one of the older practitioners present, that the asthmatic paroxysm occurs quite as frequently by

1. Bergson: Recherches sur l'Asthme, 1852.
2. Burckhardt: Ber. üb. d. Chir. Abtheilung d. Ludwig-Spitals, 1884 and 1885.
3. Shattuck: Keating's Enc. for Child. Dis.

day as by night. I would remind him that I am dealing with those subjects of asthma in whose upper air-passages some *obstructive lesion* has been demonstrated, for example, adenoids, and *not* with bronchial asthma. In the former case, I would suggest as a reason for the nocturnal attacks, or rather attacks during sleep at any time, the fact that the dry throat caused by mouth-breathing calls forth a constant, even if unconscious, effort on the part of the patient during waking hours to swallow his saliva, or something else, so that the parts may be moistened.

I cannot see why asthma is not a name for an acute exacerbation in the course of a chronic bronchitis, due to partial suffocation because of the swallowing of the tongue (to be explained later); or, if the lesion is not completely obstructive, then of sufficient extent to cause marked interference with the functions of the nose, producing dryness in the throat, which wakens the vaso-motor nerves into over-activity, causing sudden circulatory disturbance, and followed by excitability in the general nervous system, which has until now been quiescent. Why is it not somewhat the same sort of thing as a sudden awakening from sleep, or as a fright, causing dyspnea and cyanosis? This, again, is a suggestion offered for what it is worth, and for information.

I do not mean to say that the subject of adenoids must necessarily *at once* have asthma. In the interest of science, to the momentary neglect of the patient, I would be very greatly obliged to you, gentlemen, if, in responding to summons to your mouth-breathing patient, you would put your stethoscope on the upper and anterior portions of the chest, and see if a state of things akin to the asthmatic paroxysm is present.

All writers are agreed in this, that underlying the condition called asthma (except bronchial asthma), is a *neurosis*. Now, how is a neurotic temperament developed because of adenoids in the naso-pharynx?

Normally, the under jaw is held against the upper one by atmospheric pressure. When the lips are closed, the pressure of the tongue against the palate, or processus alveolaris, produces a sort of vacuum (in imitation of the suction-pump) which prevents the sinking down of the under jaw. When, in consequence of mouth-breathing, the formation of this vacuum is impossible, the under jaw sinks, and a peculiar snoring is heard during sleep. This snoring is produced, not by the soft palate, but by contact between the base of the tongue and the epiglottis; in other words, there is *a swallowing of the tongue.* (Exactly the same thing may occur in chloroform narcosis.) Thus a *stenosis* of the upper air-passages is produced; sleep is disturbed, hence not restful. The general condition suffers, and a neurotic temperament is engendered.

In noting the extent to which this neurosis may lead, in subjects of adenoid disease, Chatellier[1] cites cases of melancholia, hypochondriasis and of intense headache in the upper and back part of the head. These conditions, he says, relate to exacerbations in the course of the disease.

I am sure you will be glad to know that I do not propose to discuss the question of "hay-fever" in relation to adenoids. Within recent years, so many writers have run wild upon this subject, as to lead one, at any rate, who is opposed to reflex explosions, caused by nasal irritation, to inquire what constitutes a normal nose? I have been asked what *kind* of lesion in the nose to look for in cases of "hay-fever!" If there is no such thing as a *healthy* nose, certainly no two *diseased* noses are alike. Look for *some, any* lesion in the nose. Cases are too numerous to cite. I will merely mention that only yesterday I removed a cystic mass of material from the middle turbinated body, which mass, together with hypertrophy of both the middle and inferior turbinated bodies, completely occluded one nostril, the other being nearly closed by a deviated septum. This patient, now twenty-five years old, has been an intense sufferer with asthma (not hay-fever) since childhood, and is now in a wretched state of general debility.

In this paper I have to deal with hay-fever only in so far as it is associated with asthma. I must either withdraw my definition of asthma or bring the special treatment of my subject to a close. For I have stated that, asthma, whatever it may be pathologically, is clinically only a concomitant of emphysema and bronchitis; or, it is a desultory condition, of uncertain persistence, attendant upon the morbid state of the pituitary membrane along some portion of the upper respiratory tract. It was to "kill two birds with one stone" that I dwelt at length upon the disturbances of innervation, mechanical changes, etc., incident to adenoids and these conditions in the chest.

I wish to mention, also, the relation of flatulence, intestinal parasites, etc., to asthmatic paroxysms in subjects of adenoid disease. Prefatory to explanation, I must again acknowledge my adherence to Bosworth's opinion, that no asthmatic paroxysm,

1. Loc. cit.

aside from that of true bronchial asthma, can exist without there being *some* lesion in the upper air-passages. I do not mean that such a lesion is *alone* responsible; but, "vaso-motor weakness of the bronchial tubes," which, in the presence of unusual reflex disturbance, such as flatulence or intestinal parasites, may predispose the patient to an asthmatic attack. The flatulence is simply an adventitious symptom, or complicating circumstance, which precipitated the attack.

If the upper air-passages of the asthmatic adult were more generally examined, I believe that, in every instance, one or more *conditions* pathognomonic of previously existing adenoids would be found by inductive reasoning. I refer, for example, to the notable ("Gothic") arching of the roof of the mouth; lateral compression of the epiglottis; or chronic nasal catarrh, due to circulatory disturbances.

The arched palate is very commonly observed.

This drawing shows the lateral compression of the epiglottis, which is somewhat rare, and not to be mistaken for a lateral deflection sometimes seen in paralysis. It shows, also, how the epiglottis, coming in contact with enlarged glands at the base of the tongue, gives rise to a "tickling cough," "a *grating*," "a feeling as if something were in the upper part of the throat, which can be neither coughed nor hawked out." The subject of this sketch, referred to me by the kindness of Dr. Gilray, presented enlarged follicles upon the pharynx, and enlarged glands at the base of the tongue. I very commonly find these enlarged glands as a concomitant of follicular pharyngitis. I might mention, in this connection, hypertrophy of the faucial tonsils, and those cases of disease of the several air-sinuses in the adult, as evidence of preëxisting obstruction in the upper air-passages. I do not believe, however, that hypertrophied faucial tonsils alone have much, if anything, to do with respiration.[1,2] Deformities of the jaws of the adult, and of the progressing deformity of the jaws and alveolar processes in young children, (both results of forced breathing,) persisting in spite of the dentist's effort to overcome atmospheric pressure by means of his plates, has been thoroughly studied by Dr. Hooper.[3]

(The question of adenoids and other obstructive lesions of the upper air-passages in relation to deaf-mutism, will be the subject

1. Dupuytren: (Rep.) d'Anat., 1828.
2. Robert: Hyp. of the Tonsils, etc., 1843.
3. Hooper: The Mech. Effects of Aden. Veg. in Children. Rep. of Boston City Hospital, Fourth Series. 1889.

of a paper by me as soon as opportunity offers for careful investigation.)

As to tabulation for age of occurrence, I consider the subject of "adenoid asthma," if I may coin a term, altogether too new for such statistics to be of much value. Salter[1] declares the first decade to be the age of prevalence. The greatest number of Bosworth's[2] cases occurred in the third decade; while Jurasz[3] finds his statistics increased between the first and second decades.

When I tell you that the first operation done in Boston for the radical cure of this particular respiratory obstruction, was at the hands of Dr. Hooper as late as 1885; when, too, you look at the length of the chapter in your text-books, published as late as five years ago, on asthma in children, (in which, by the way, nothing is said about congenital asthma,) you will appreciate why this whole subject is still in its infancy. That we shall know a good deal more about it in the near future will be due largely to inquiry on the part of the medical practitioner.

In conclusion upon the theme, *per se*, of this paper, I have to thank you, gentlemen, for your close attention. As this is the first time I have had the pleasure of appearing before this section, I would ask your further indulgence for a few minutes, at the risk of being technical and surgical, that so I may put myself on record in Buffalo as standing for a few points in relation to the general subject.

The excuse generally offered by the specialist in my department for a paper before medical clinicians is, first, that the general medical practitioner should be told how to recognize the presence of adenoids; secondly, that he should operate the cases in which he has made the diagnosis. Now the general medical practitioner does not usually examine with the mirror, but with the finger. During my limited experience in teaching, I have told the physician, or student, that by digital examination in a given case, he would find adenoids. Almost invariably he has confirmed the suggested diagnosis. Telling him, now, to examine the same case with the rhinoscope, he finds no suspicion of disease. The fault lies in this, that digital exploration causes contraction of the soft palate; also the rugæ of the mucous membrane are brought into undue prominence. A normal condition is, therefore, pardonably mistaken by the inexperienced for an abnormal one. Operation under these circumstances would be most deplorable.

---

1. Loc. cit.
2. Loc. cit.
3. Loc. cit.

I would not be understood that digital examination is of no value. The lateral and postero-superior regions of the vault of the pharynx are not readily seen, if seen at all, in the mirror. This method is also of great topographical value, as well as for determining differences in density of the tissue. I never operate without supplementing rhinoscopic by digital examination.

Anyone, with a little practice, can get a view of the larynx in most cases. The rhinoscopic image is, unfortunately, not so easily obtained. Having had considerable experience with cases in which the use of the rhinoscope has led the way to almost immediate recovery from a diagnosis of anemia, or even of incipient phthisis, I would earnestly urge you to more careful practice in the use of this instrument. Dr. Hooper[1] says: "With a tithe of the attention paid to the naso-pharyngeal cavity, which is given to other less important regions of the body, there would be no necessity for referring such cases to the specialist for either diagnosis or treatment."

Allow me to note a few points which you will find of value in the practice of rhinoscopy. Where the difficulty is with the arched tongue, any undue force with the tongue depressor will be of no assistance. Most children old enough to understand what is wanted of them, can very soon train themselves to flatten their tongue. Loewenberg[2] recommends the inclination of the thorax forward and the head backward. Thus the greatest distance between the soft palate and the back of the throat is obtained, by the pushing back of the cervical vertebræ.

I put my patient in an upright position, then draw him towards me, without his moving in the chair. The head is then tipped backwards.

Another method which often succeeds, is to have the patient sit upright and, without inclining the thorax, to tell him "*to drop his chin.*"

A very young child understands, in the rule, what you mean when, with the mirror successfully in position, you tell him to breathe through his *nose.* This is another important aid, impossible, of course, if there be considerable stenosis.

As to operation by the general medical practitioner—unless, indeed, he has had sufficient experience to possess special knowledge—I wish most emphatically to say that, of all the naso-

1. Loc. cit., No. 1.

2. Loc. cit., No. 1.

laryngeal conditions commonly requiring operation, I regard this one most eminently fit to be sent to the specialist. It is not by any means a difficult matter seriously to wound the underlying healthy parts. The profuse hemorrhage which I have so often seen in forceps operations in unskilled hands for removal of this tissue, is undoubtedly explained in this way. With the instrument to be explained further on, such an accident would be unpardonable. (As a matter of fact, I get very little hemorrhage in my adenoid operations; and what there is, usually ceases before recovery from chloroform.)

To say nothing of a lack of knowledge of the regional anatomy and relative thickness of the membrane, the favorite instrument in the hands of the general medical practitioner is usually the finger-nail. Such an operation I regard as ridiculous. If surgical interference is demanded, it seems to me that a *surgical instrument* should be used. The "finger-nail" procedure is also entirely at variance with antiseptic surgery. My school days are not so far remote, that I cannot remember noting in a lecture by Dr. Fitz, Shattuck professor of pathology in Harvard University, the statement that, to make the tips of the fingers aseptic is an impossibility. (The finger tip to *feel* with is a different matter from the same thing to *scratch* with.) Moreover, the result is never complete at one sitting. I searched in vain for condemning authority until I found B. Fränkel[1] stating that he had never followed this advice; and that "in view of the many specially adapted instruments of the present day, to use the finger-nail would seem like returning to the stone-age of our scientific knowledge." Many of you will have read, in so recent a work as Keating's "Encyclopedia of Children's Diseases,"[2] that "this method is by far the most effective and is without any possible objection." You will also find these growths defined in the same article as *papillomatous*. This latter statement, as already proved, makes the former valueless for authority. Bosworth[3] had evidently omitted this article in his research for authority to state that "no one but Woakes still adheres to this idea of the papillomatous nature of these growths."

As to the *best* instrument for operating, I have simply to say, every man to his own choice. Books and monographs in English

1. Loc. cit.
2. Harrison Allen: Adenoid Growths of the Vault of the Pharynx.
3. Op. cit.

mention various kinds of *curettes.* It is the exception that any form of curette is advised as preferable to forceps in some form. The Gottstein ring-messer is the instrument in most general use in Germany. Several years ago I began an operation with it, only at once to call for my forceps. If this same Gottstein ring-messer had not been made, I might not have the instrument which has given me, and many others who possess it, the utmost satisfaction. I have not asked Dr. Lefferts' permission to publish this picture ; but, as the instrument has largely superseded the original, even in Germany, and is so highly prized by many operators, I do not know that his modesty in withholding more general information about his instrument is altogether becoming. Its special adaptability is in the tipping toward the handle of the upper part of the curette. It is but necessary to consider where the cutting edge of an ordinary right-angled curette (*e. g.* Hartmann's) would reach the vault of the pharynx, remembering the limited downward obliquity of the shank, when the instrument is in position, because

THE GOTTSTEIN RING-MESSER, MODIFIED BY LEFFERTS.

of its contact with the lower jaw. This projection towards the handle brings the cutting edge of the curette as far forward as the septum. Indeed, with an upper corner of the curette, I have scraped away portions of the growth which have encroached upon the lumen of the choanæ. The removal with the finger-nail (so commonly done) of ragged fragments left behind in forceps operations, is rendered entirely unnecessary when this instrument is used. It makes a *clean sweep !* I can *feel* with it as with a probe, which is impossible, I think, with the forceps. With the currette in position, I use force sufficient to crush through the growths. Maintaining this force, the handle of the instrument is elevated, beginning, at the same time, the downward sweep with the cutting edge. (At a recent operation, my assistant, holding the head, told me that he felt the initial *crushing*, the impulse being communicated through the bones of the head.)

But I am afraid, gentlemen, that my enthusiasm, expressed at any further length, would cast doubt upon the philosophical truism that selfishness and generosity are synonymous terms.

My thanks are due to Dr. E. J. Gilray for reviewing a number of monographs in English on this subject.

21 WEST NORTH STREET.

[NOTE.—The Gottstein ring-messer, modified by Lefferts, is made by Pfau, in Berlin, and Rynders, New York.]

## Figures and Tables

**Figure f1:**